# Prediction of Expected Fouling Time During Transmembrane Transition in Reverse Osmosis Systems

**DOI:** 10.3390/membranes15060170

**Published:** 2025-06-06

**Authors:** Jozsef Lakner, Gabor Lakner

**Affiliations:** Hidrofilt Water Treatment Ltd., Magyar u. 191, H-8800 Nagykanizsa, Hungary; lakner.jozsef@arek.uni-obuda.hu

**Keywords:** reverse osmosis, membrane fouling, fouling time prediction, probabilistic modelling, normal distribution, maintenance optimization, cost estimation

## Abstract

Membrane filtration, including reverse osmosis filtration, is widely applied in water treatment worldwide, offering solutions to a broad range of separation challenges. However, due to the porous structure of membranes, they are prone to fouling, which reduces their efficiency and can eventually render the membranes incapable of functioning. In such cases, a systemic intervention becomes necessary, highlighting the importance of accurately predicting the expected fouling time. Various approaches for estimating fouling processes and times are well documented in the literature. However, a common limitation of these methods is that they typically assume constant and well-defined operating parameters over time. Under such stable conditions, the process can be described deterministically, and the fouling time can be predicted using straightforward extrapolation techniques. However, in industrial practice, process conditions often fluctuate due to multiple influencing factors, making fouling time a variable quantity. Therefore, it can be more appropriately treated as a random variable characterized by a mean value and standard deviation. Rather than predicting a precise fouling time, it is more relevant to define a probabilistic interval within which the fouling is expected to occur with a specified confidence level (e.g., 95%). The associated maintenance scheduling can then be optimized based on economic criteria. The probability-based model presented herein defines this interval based on operational measurements, thereby providing users with a time window during which maintenance should be planned. From this point forward, the exact timing of interventions becomes a matter of technical feasibility and economic optimization.

## 1. Introduction

Rapid urbanization and industrialization in recent decades have resulted in a significant increase in wastewater containing various contaminants [1]. The discharge of untreated wastewater into the environment poses serious risks to ecosystems, living organisms [2], and human health [3]. Therefore, effective treatment methods are required that ensure fast operation and high efficiency while maintaining relatively low energy consumption. Membrane-based water treatment technologies can meet all of these criteria [4,5].

**Figure 1:**
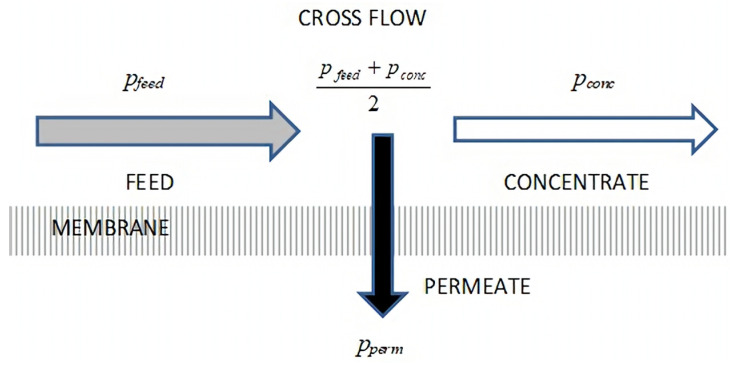
The cross-flow membrane filtration process.

According to internationally accepted terminology, a membrane is defined as a selective barrier between two phases [6]. It separates a multi-component feed stream into two main fractions: the permeate, which is the portion that passes through the membrane, and the retentate (or concentrate), which is the portion retained by it. A schematic representation of this process is shown in Figure 1.

From the perspective of driving force, membrane processes can be classified into several types [6,7]. In water and wastewater treatment, processes that primarily rely on pressure difference as the driving force—such as microfiltration, ultrafiltration, nanofiltration, and reverse osmosis—are particularly important [7]. These pressure-driven membrane processes are applied for a wide range of purposes, including the following:

- Degassing, such as the removal of ammonia [8,9];
- Separation based on molecular size or mass, such as seawater desalination [10,11];
- Liquid–liquid separation, such as oil-contaminated water treatment [12];
- The removal of pharmaceutical residues [13];
- The retention of bacteria and viruses [14].

Fouling plays a critical role in the operation of membrane systems, as it often necessitates intervention—either in the form of maintenance or membrane replacement. Such interventions incur costs and require organizational planning; therefore, it is essential to estimate the timing and extent of fouling in advance. This allows for cost planning and the scheduling of regular maintenance operations.

Recent studies have highlighted the complexities of membrane fouling in reverse osmosis systems and the need for advanced predictive models. For instance, a comprehensive review by AlSawaftah et al. [15] discusses various fouling mechanisms and emphasizes the importance of accurate prediction and mitigation strategies. Similarly, research by Liu et al. [16] explores the influence of ultrafiltration pretreatment on RO membrane fouling, underscoring the role of pretreatment processes in fouling control. These studies underscore the necessity for robust statistical models that can predict fouling behaviour under varying operational conditions.

Models available in the literature (see Section 2) describe fouling as an analytical function of time. However, these models rely on the assumption that the parameters influencing fouling are well known and remain constant throughout the process.

In industrial applications, these conditions cannot always be guaranteed. On the one hand, some of the parameters that influence fouling time may be unknown; on the other hand, their values can fluctuate throughout the process. As a result, the relationship between fouling and the evaluated parameter (typically, operating time) must be expressed in a stochastic form using probabilistic (i.e., mathematical–statistical) methods.

In this case, fouling time becomes a random variable characterized by a probability distribution with a defined mean and standard deviation. Consequently, at any given time, the degree of fouling can only be described with a certain level of probability, making the estimation of the expected intervention time inherently uncertain.

This uncertainty can have significant cost implications, so the optimal timing of the intervention is determined by minimizing the expected cost.

The novelty of the present work lies in the integration of both technical and economic factors when determining the expected fouling time and planning the intervention strategy.

## 2. Theoretical Framework (Analytical Model of Membrane Fouling)

### 2.1. Resistivity

Material is transported through the membrane by diffusion [17]. The flux through the membrane is directly proportional to the driving force (*TMP*, transmembrane pressure) and inversely proportional to *η*, the viscosity of the mixture to be separated, and the total resistance *R*_tot_ of the system, and this flux is calculated using the following equation:

(1) $$J=\frac{TMP}{\eta R_{\mathrm{tot}}}.$$

In Equation (1), *R*_tot_ is the (total) resistance of membrane and consists of three parts, as follows:

*R*_tot_ = *R*_memb_ + *R*_pol_ + *R*_foul_, (2)

where *R*_memb_ is the resistance of the membrane material; *R*_pol_ is the resistance of the polarisation layer; *R*_foul_ is the resistance originating from membrane fouling [18]. Ideally, only the membrane material exerts resistance against the flux, but, in reality, other parameters can also act against it, for example, concentration polarisation, the fouling of the pores, and the formation of a gel layer on the membrane surface. The most important factor among these factors is fouling.

### 2.2. Membrane Fouling

Membrane fouling can occur for various reasons, so there can be several types of membrane fouling [19]. Materials that cannot pass through the membrane may be adsorbed on the pore walls, even blocking them. Additionally, fouling can occur due to the accumulation of materials on the feed (raw water) side of the membrane, forming the so-called filter cake [20]. Biofouling is a special case of membrane fouling, where various microorganisms adhere to the membrane surface, leading to the formation of a biofilm. This decreases material flow and can even alter the membrane material, ultimately shortening the membrane’s useful life [21]. This decreases the efficiency of the separation process, which may even come to a complete stop, requiring cleaning or membrane replacement to restart. Both procedures incur significant additional costs. One possible way to mitigate this effect is by using antifouling membranes [22], which can reduce cleaning frequency and extend the membrane’s useful life [23].

As a result of studies aimed at reducing fouling, the concepts of critical flux and sustainable flux were defined [24]. The former refers to the flux at which membrane fouling has not yet begun, while the latter represents a flux level that remains acceptable for the operator, even though some fouling occurs. The actual flux and the extent of membrane fouling depend heavily on the operating conditions of the membrane module [25].

If membrane fouling does occur, appropriate treatment is required [26,27], which may include regular rinsing, backwashing, and shaking of the membrane (hydraulic/mechanical cleaning), ultrasound irradiation, increasing the membrane temperature, etc. If the flux cannot be restored to the required level using these methods, the membrane must be chemically cleaned or, in extreme cases, even replaced. The choice of cleaning interval is itself a compromise; longer regeneration times can improve efficiency, but extending them too much may adversely affect system operation [28].

### 2.3. Fouling Kinetics

During fouling, the membrane resistance changes with time, i.e., *R*_tot_ *= R*(*t*), and correspondingly the flux, is dependent on time in the case of a given transmembrane pressure (*TMP)*, *J* = *J*(*t*). Four basic models are used for assessing the time dependence of the flux, considering the type of fouling: complete pore fouling, internal pore fouling (standard), partial pore fouling, and cake filtering [29].

In the case of a given flux (*J* = const.), the transmembrane pressure required for maintaining the flux depends on the time, *t*, and the temperature, *T*, and based on Equation (1), it is expressed as follows:

(3) $$TMP(T,t)=J\eta (T)R_{\mathrm{tot}}(t),$$

where *η*, the viscosity of the liquid (water), is a function of the temperature due to the so-called Arrhenius-type [30] temperature dependence [31], which is calculated using the following equation:

(4) $$\eta (T)=\eta_{0}e^{-\frac{E_{\eta }}{RT}},$$

where *E_η_* is the activation energy (for water: *E_η_* = 16 kJmol^−1^), *R* = 8.84 Jmol^−1^K^−1^ is the gas constant, *T* is the temperature of the liquid (K), and *T*_0_ = 293 K (20 °C) is the standard temperature. Substituting Equation (4) into Equation (3), the transmembrane pressure for the given flux (*J*) as a function of temperature:

(5) $$TMP(T,t)=J\eta_{0}e^{-\frac{E_{\eta }}{RT}}R_{\mathrm{tot}}(t).$$

The above equation is also an Arrhenius-type correlation.

Transmembrane pressure as a function of time, *t*, can be derived for different fouling types from the flux on the basis of Equation (1). A linear approximation of *TMP* is shown in the next equation:

(6) $$TMP(t)=TMP_{0}+at.$$

The above equation seems to be sufficient for the determination of fouling time (see below).

### 2.4. Fouling Time

At a given temperature *T*, the transmembrane pressure (*TMP*) is directly proportional to the total membrane resistance, *R*_tot_. If fouling is complete, then $R_{\mathrm{tot}}(t\to \infty )$; therefore, TMP(t→∞), which also approaches infinity. In practice, however, complete fouling is never observed, so only partial (percentage-based) fouling can be defined, which is expressed by the following dimensionless ratio:

(7) $$\frac{R_{\mathrm{tot}}(t=t_{\mathrm{foul}})}{R_{\mathrm{tot}}(t=0)}=1+X_{\mathrm{foul}}.$$

Equation (7) allows for the definition of various fouling levels, each corresponding to a specific fouling time *t*_foul_. For example, different thresholds can be associated with the following:

- Simple chemical cleaning;
- Intensive washing;
- Full membrane replacement.

If we select *X*_foul_ = 0.25, this means that the fouling time *t_foul_* is defined as the time at which the membrane resistance increases by 25% relative to its initial value.

Although choosing a 25% increase in *TMP* as the intervention threshold may appear arbitrary, it is in fact supported by the standardized guidelines of major membrane manufacturers. For example, according to DuPont’s cleaning guidelines for FilmTec™ membranes, cleaning is recommended when the pressure drop increases by 15%, as higher pressure drops may result in irreversible membrane damage [32]. Similarly, Toray recommends initiating cleaning for its SU-720R brackish water RO membranes when the pressure drop reaches 1.5 times the initial value, corresponding to a 50% increase [33]. These guidelines support the industry-wide recommendation of initiating membrane cleaning when the transmembrane pressure (*TMP*) increases by 10–30% for the following reasons:

- Maintain membrane efficiency;
- Avoid irreversible damage;
- Prevent operational disruptions.

Our own practical experience confirms that a 25% increase in *TMP* is a reliable and effective threshold for initiating intervention, as it consistently correlates with performance loss requiring maintenance.

Since *TMP* is directly measurable, we can use Equation (3) to relate it to membrane resistance at a given temperature. The ratio of *TMP* under fouling conditions to its initial value is expressed as follows:

(8) $$\frac{TMP_{\mathrm{foul}}}{TMP_{0}}=\frac{R_{\mathrm{tot}}(t=t_{\mathrm{foul}})}{R_{\mathrm{tot}}(t=0)}=1+X_{\mathrm{foul}},$$

This implies that resistance-based fouling can be monitored simply by measuring the transmembrane pressure.

Taking *t* = *t*_foul_ in the previously defined linear resistance growth model (Equation (6)), the transmembrane pressure at the fouling point is expressed as follows:

(9) $$t_{\mathrm{foul}}=\frac{TMP_{\mathrm{foul}}-TMP_{0}}{a},$$

where *TMP*_0_ is the transmembrane pressure at time *t* = 0, corrected for temperature, and *a* is the constant fouling rate. This expression forms the basis for determining the fouling time under realistic operating conditions, using measurable system parameters.

## 3. Experimental Part

### 3.1. The Equipment

The equipment used in this study is an HF-RO-0.7-type brackish water reverse osmosis (RO) system, manufactured by Hidrofilt Water Treatment Ltd. (Magyar Str. 191, Nagykanizsa, Hungary), and integrated into a boiler feed water treatment process. The system operates with a two-pass reverse osmosis setup, followed by electrodeionization (EDI) for final polishing.

**Figure 2:**
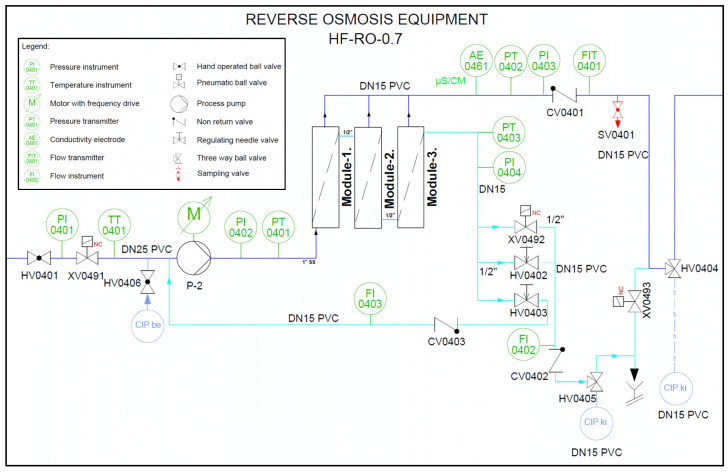
The process and instrumentation diagram (P&ID) of the HF-RO-0.7 reverse osmosis system—the second-pass unit configuration used in the study. The blue line in the diagram represents the feedwater to the reverse osmosis (RO) system, which is connected to the RO permeate line (the product water line). The turquoise line indicates the RO concentrate line, which carries the wastewater (concentrate) that remains after the filtration process.

The water source is municipal, and the conventional pretreatment includes a softener to reduce hardness and an activated carbon filter to remove residual chlorine from the RO feed water. The data used in this study were obtained from the second-pass RO in this system, meaning that, in this case, the feed water of this unit is the permeate from the first-pass RO. The flow rates in the second-pass system are as follows: inlet: 1000 L/h; permeate: 700 L/h; concentrate: 200 L/h; and recirculation: 100 L/h. The following process parameters were monitored: temperature, permeate and concentrate flow rates, recirculation flow rate, inlet, permeate, and concentrate pressures, and permeate conductivity. All instruments are integrated into a PLC-controlled automation system, which includes a data logging module for continuous data acquisition. The configuration of the reverse osmosis system is shown in Figure 2 (process and instrumentation diagram—P&ID).

### 3.2. Measurement Results

**Figure 3:**
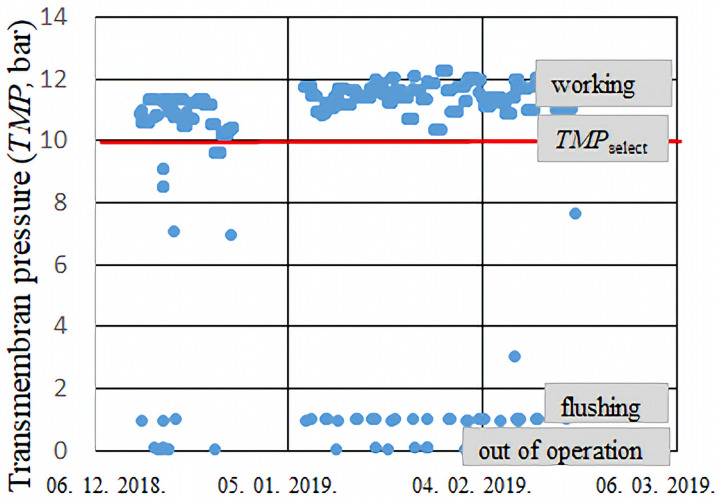
Transmembrane pressure *TMP(T)* values as a function of time (date), with a marked selection limit, *TMP*_select_, indicating different operating modes, such as ‘working’, ‘flushing’ and ‘out of operation’.

During the examination, on the input part of the membrane, the *p*_feed,*i*_ feed pressure, *p*_conc,*i*_ output (concentrate) pressure, and *p*_perm,*i*_ cross-direction (permeate) pressure as well as the temperature were measured as a function of time (the number of measurements, *i*), specified by date. Additionally, conductivity and flux rate measurements were taken (flux *J*). We managed to keep the latter at 700 ± 3 litre/hour with relative accuracy. The measured values of transmembrane pressure (*i*th measurement) are as follows:

(10) $$TMP_{i}(T_{i})=\frac{p_{\mathrm{feed},i}+p_{\mathrm{conc},i}}{2}-p_{\mathrm{perm},i},$$

where *T = T_i_*, since the temperature can vary from measurement to measurement (discussed in Section 3.3), and the pressures (*p*’s) correspond to those shown in Figure 1. The transmembrane pressure (*TMP*) as a function of time (day, month, year) is plotted in Figure 3, with markers indicating periods when the system was either not operational or undergoing cleaning. The total number of measurements was approximately 4500. The entire measurement period lasted 75 days, corresponding to a measurement taken roughly every 30 min.

### 3.3. Sample Selection

A few data points that significantly deviated from the expected trend were removed. For this purpose, a threshold value, referred to as *TMP*_select_ value was defined. The excluded samples primarily corresponded to periods when the system was either not operational or undergoing maintenance—the drawn line (Figure 3).

Statistical evaluations require independent samples. Consecutive measurements were considered independent only if their values differed, i.e., $TMP_{i}\neq TMP_{i+1}$. Specifically, when all process parameters remain unchanged between two successive measurements (i.e., the measurements are not independent), the *TMP* increases by approximately 0.0005 bar. If the difference is greater than this value, at least one operating parameter must have changed, and the two measurements can therefore be considered independent. The larger the difference, the more certain this independence becomes.

Since the measurement accuracy is 0.01 bar, any observed difference exceeding this threshold strongly supports the assumption of independence. If no such difference was found, the subsequent sample(s) were excluded from further analysis. As a result of this filtering process, 104 corrected independent samples were obtained.

The nominal temperature was defined as *T*_0_ = 20 °C (293 K), while the actual temperature fluctuated between 18.2 °C and 24.1 °C. Since the transmembrane pressure is affected by the temperature of the liquid, these fluctuations required compensation. The compensation was performed relative to the nominal temperature, *T*_0_ = 293 K (20 °C), which is considered the standard reference temperature.

According to Equation (5), the temperature dependence of the transmembrane pressure [30] is due to the temperature dependence of viscosity, which can be expressed for a given temperature *T_i_* in the following form [31]:

(11) $$\eta (T_{i})=\eta_{0}e^{-\frac{E_{\eta }}{RT_{0}}}e^{-\frac{E_{\eta }}{R}\left(\frac{1}{T_{t}}-\frac{1}{T_{0}}\right)}=\eta (T_{0})[1+0.0224(T-T_{0})].$$

**Figure 4:**
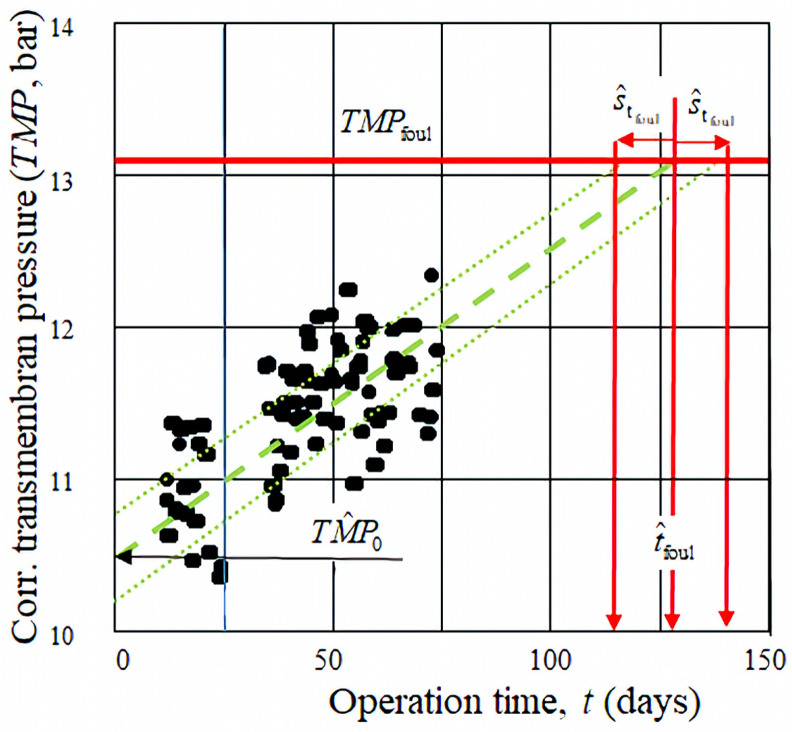
The corrected *TMP* data (from Figure 3) plotted as a function of time (days) after cleaning and temperature normalization. This figure includes the linear regression function (Equation (6)), and the lines represent ${\hat{s}}_{t_{\mathrm{foul}}}$, standard deviation, as well as the fouling threshold transmembrane pressure, *TMP*_foul_. Green lines represent the linear regression and show the trend of the data points. Red lines represent the fouling threshold transmembrane pressure (*TMP*_foul_.), an important reference value to monitor the system’s performance. Blue line indicates the initial state (*TMP*_0_), representing the starting value of the measurements.

The above equation is a first-order approximation. Substituting values of *E_η_* = 16 kJmol^−1^, *R* = 8.84 Jmol^−1^K^−1^, and *T*_0_ = 293 K (20 °C), the following transmembrane pressure, corrected for a temperature of 20 °C, can be defined as follows:

(12) $$TMP_{i}=\frac{TMP_{i}(T_{i})}{\left[1+0.0224(T_{i}-T_{0})\right]},$$

which can now be considered as the measured values of the process according to the first-order (isotherm) kinetics of Equation (6). The cleaned values of the samples, compensated with the temperature (*TMP*), are presented in Figure 4 as a function of time, *t* (of the samples number, *j*, 20≤j≤104).

## 4. Probability Model

When fouling kinetics are determined under laboratory conditions, the parameters can be carefully selected and controlled. In such cases, the fouling time can be estimated using simple extrapolation, also referred to as point-based estimation. In contrast, in industrial settings, the parameters may fluctuate over time. As a result, both these parameters and the resulting fouling time must be treated as random variables, characterized by a mean value, standard deviation, and a probability distribution function.

### 4.1. Linear Regression

Fouling is a time-dependent process characterized by a gradual increase in the temperature-corrected transmembrane pressure (*TMP*). The fouling time *t*_foul_ is defined as the moment when the *TMP* reaches a predefined critical threshold, *TMP*_foul_.

According to Figure 4, there is a stochastic relationship between the transmembrane pressure (*TMP*) and the treatment time *t*, as also noted in [34]. Therefore, the fouling time (*t*_foul_) must be determined using mathematical and statistical methods, which have been successfully applied in our previous studies [35,36].

The values of the parameters in Equation (6) were estimated from a set of *j* measurements, according to the following expressions [37]:

(13) $$a(j)=\frac{j\sum_{i=1}^{j}TMP_{i}t_{i}-\left(\sum_{i=1}^{j}TMP_{i}\right)\left(\sum_{i=1}^{j}t_{i}\right)}{j\sum_{i=1}^{j}t_{i}^{2}-{\left(\sum_{i=1}^{j}t_{i}\right)}^{2}},TMP_{0}(j)=\frac{\left(\sum_{i=1}^{j}TMP_{i}\right)\left(\sum_{i=1}^{j}t_{i}^{2}\right)-\left(\sum_{i=1}^{j}t_{i}\right)\left(\sum_{i=1}^{j}TMP_{i}t_{i}\right)}{j\sum_{i=1}^{j}t_{i}^{2}-{\left(\sum_{i=1}^{j}t_{i}\right)}^{2}},\;$$

where *TMP_i_* is the value of the temperature-corrected transmembrane pressure at the *i*-th measurement, *j* is the total number of measurements, *i* is the index of each measurement (*i* = 1, 2, …, *j*), and *t = t_j_* and *t_i_* are the corresponding measurement times. To some extent, both parameters in Equation (13)—namely the fouling rate *a*(*j*) and the initial transmembrane pressure *TMP*_0_(*j*)—may depend on the number of measurements *j*. Consequently, the transmembrane pressure function described by Equation (6) also becomes a function of *j* through these parameters. Thus, the estimated value of *TMP* at a given time *t*, calculated using Equation (6) together with the parameter estimates from Equation (13), may vary depending on the sample size *j*. This dependency is illustrated in Figure 4, where the black line corresponds to the case of *j* = 104 measurements at *t* = 75 days.

### 4.2. Standard Deviations of Parameters

The parameters in Equation (6) are estimated from *j* measurements, and the actual values fluctuate around these estimates. The magnitude of this fluctuation is described by the standard deviations of the regression parameters, which are given as follows [36,37]:

(14) $$s_{a}(j)=\sqrt{\frac{\mathrm{Rez}}{(j-2)\cdot \sum_{i=1}^{j}{\left(t_{i}-\bar{t}\right)}^{2}}},s_{{\mathrm{TMP}}_{0}}(j)=\sqrt{\frac{\mathrm{Rez}\cdot \sum_{i=1}^{j}t_{i}^{2}}{j(j-2)\cdot \sum_{i=1}^{j}{\left(t_{i}-\bar{t}\right)}^{2}}}.$$

Here, $\mathrm{Rez}=\sum_{i=1}^{n}{\left[TMP_{i}-(\hat{a}t_{i}+T\hat{M}P_{0})\right]}^{2}$ denotes the residual sum of squares—that is, the sum of squared differences between the measured and predicted transmembrane pressure values. The variable $\bar{t}$ is the mean measurement time, i.e., the average of all time values *t_i_* corresponding to the *j* measurements.

The standard deviation of the transmembrane pressure at a given time $t\leq t_{j}$ (*j.* measurement), based on the rules for error propagation in linear regression, is expressed as follows [33]:

(15) $$s_{\mathrm{TMP}}(t,j)=\sqrt{{\left[s_{a}t\right]}^{2}+{\left[s_{{\mathrm{TMP}}_{0}}\right]}^{2}}.$$

This value depends on both the time *t* and the number of measurements *j*. In the case of $s_{a}^{2}t_{j}^{2}<s_{{\mathrm{TMP}}_{0}}^{2}$, Equation (15) can be approximated by the following expression:

(16) $$s_{\mathrm{TMP}}(t,j)≅s_{\mathrm{TPM}}(t=t_{j},j)=s_{\mathrm{TMP}}(j).$$

The value from the above equation is constant and only depends on *j*, $s_{\mathrm{TMP}}≅s_{\mathrm{TPM}}(j)$. Accordingly, alongside the regression line defined by Equation (6), two additional lines—belonging to the two standard deviation values—can be plotted, representing the confidence band of the *TMP* prediction. This confidence band is expressed as follows:

(17) $$TMP^{\pm }(t)=at+TMP_{0}\pm s_{\mathrm{TMP}}.$$

This confidence band also depends on the number of measurements j over the time interval considered, as shown in Figure 4.

### 4.3. Fouling Time—Point Estimation

The estimated value of the transmembrane pressure at the fouling point, based on Equation (9), is given as follows:

(18) $${\hat{t}}_{\mathrm{foul}}(j)=\frac{TMP_{\mathrm{foul}}-TMP_{0}}{a},$$

Here, *a* and *TMP*_0_ are calculated using Equation (13), and may vary slightly depending on the number of measurements *j*. Thus, the estimated fouling time derived from Equation (18) may also depend on *j*.

The standard deviation of the fouling time, considering the propagation of uncertainty from Equations (15) and (16), is given by the following equation:

(19) $${\hat{s}}_{t_{\mathrm{foul}}}(j)≅\frac{s_{\mathrm{TMP}}}{a},$$

as ${\hat{s}}_{\mathrm{TMP}}(t_{\mathrm{foul}})=s_{\mathrm{TPM}}$ from Equation (16), and it also depends on *j* as the above equation.

The definition of *TMP*_foul_ depends on the chosen fouling criterion. In practice, a membrane is typically considered fouled when the transmembrane pressure exceeds the initial pressure (*TMP*_0_) by approximately 25% (See Section 2.4). This threshold is widely accepted in the membrane industry.

Therefore, Equation (18) provides a point estimate of the fouling time, while Equation (19) gives the standard deviation of this estimate. However, in practical applications, it is also important to provide an interval estimate—a confidence interval that defines the range around the estimated value within which the actual fouling time falls with a given probability (e.g., 95%).

To construct such an interval, the distribution function of the estimated variable must be known—typically assumed to follow a normal distribution.

### 4.4. Normal Distribution Approximation and Fouling Interval

The transmembrane pressure values, *TMP_i_*, corresponding to specific time points, *t_i_*, are influenced by a large number of independent parameters, each contributing only slightly to the overall value. As a result, the conditions for applying a normal distribution are satisfied, and the fouling time can be treated as a random variable that follows a normal distribution. Consequently, the probability of the fouling time falling within a given interval can be described using the properties of the normal distribution, as shown below [38]:

(20) $$P\left\{t_{\mathrm{foul}}<t\right\}=\Phi \left\{\frac{t-{\hat{t}}_{\mathrm{foul}}}{{\hat{s}}_{t_{\mathrm{foul}}}}\right\}.$$

In Equation (20), Φ is the standardised distribution function of the normal distribution with a mean value of ${\hat{t}}_{\mathrm{foul}}$ (estimated) and a standard deviation of ${\hat{s}}_{t_{\mathrm{foul}}}$.

The validity of the normal distribution assumption can be assessed using a goodness-of-fit test, such as the Chi-square (*χ*^2^) test, which was performed using Microsoft Excel. However, a limitation arises from the fact that a given time point *t_i_* typically corresponds to only one (or occasionally a few) *TMP_i_* measurements, whereas the (*χ*^2^) test requires a larger sample size per class. To overcome this limitation, we define a new random variable: *τ_i_ = t_i_* − *TMP*(*t_i_*), which has an expected value of zero. We further assume that the distribution of *τ_i_* is identical for all *i*, i.e., *τ_i_* = *τ*. If τ follows the normal distribution, then the fouling time *t*_foul_ also follows a normal distribution. Under this transformation, the sample size becomes 104, which is sufficient for conducting a statistical test. The result of the (*χ*^2^) goodness-of-fit test yielded a test statistic of $\chi^{2}=22.5$, which is lower than the critical value at the 95% confidence level, $\chi_{0.95(>100)}^{2}=77.9$. Therefore, the assumption that fouling time follows a normal distribution is accepted at the 95% confidence level.

Based on the assumption of normal distribution, the fouling time can be defined with a given probability level, **P**. At the theoretical extremes, if **P** = 0, fouling certainly does not occur, and the corresponding time *t*(**P** = 0) approaches negative infinity. Conversely, if **P** = 1, fouling certainly occurs, and the associated time *t*(**P** = 1) approaches positive infinity. However, in reality, the fouling time *t* is finite, and the probability lies within the open interval 0 < **P** < 1. Therefore, it is necessary to define a time interval within which fouling is expected to occur with a given confidence level. In practical applications, this is typically represented by a two-standard-deviation confidence interval (±2σ) around the mean fouling time, corresponding to a 95% probability level. This interval is given by the following equation:

(21) $$P\left\{{\hat{t}}_{\mathrm{foul}}-2{\hat{s}}_{t_{\mathrm{foul}}}<t_{\mathrm{foul}}<{\hat{t}}_{\mathrm{foul}}+2{\hat{s}}_{t_{\mathrm{foul}}}\right\}=0.95,$$

which means that the fouling time, $t_{\mathrm{foul}}$, falls within this interval with 95% probability.

Referring to Equation (21), the probability $P\left\{t_{\mathrm{foul}}<{\hat{t}}_{\mathrm{foul}}-2{\hat{s}}_{t_{\mathrm{foul}}}\right\}$ = 0.025 (i.e., 2.5%) is practically negligible. Therefore, a lower bound for the fouling time can be defined and denoted as ${\hat{t}}_{\mathrm{foul}}^{-}$. Conversely, if the time *t* exceeds the upper bound $t_{\mathrm{foul}}>{\hat{t}}_{\mathrm{foul}}+2{\hat{s}}_{t_{\mathrm{foul}}}$, then the probability of fouling is higher than 0.975 (97.5%), indicating that membrane fouling has almost certainly occurred. The corresponding upper limit is ${\hat{t}}_{\mathrm{foul}}^{+}$, and according to this, the interval $\left[{\hat{t}}_{\mathrm{foul}}^{-};{\hat{t}}_{\mathrm{foul}}^{+}\right]$ can be considered as the estimated fouling range.

**Figure 5:**
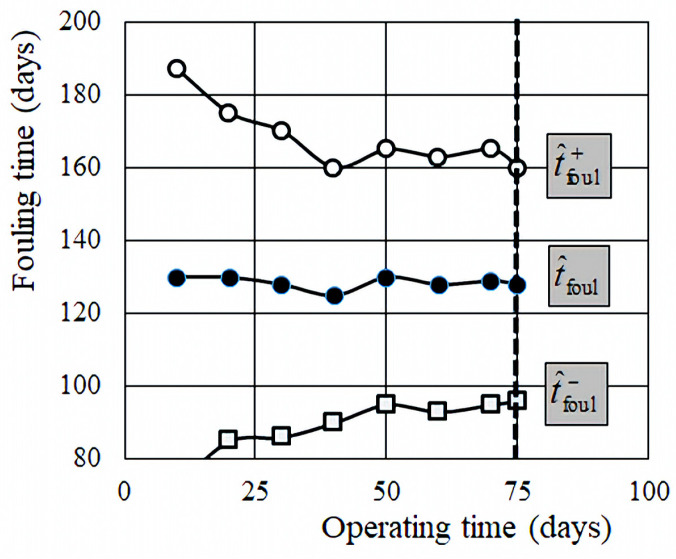
The extrapolated values, ${\hat{t}}_{\mathrm{foul}}$, of fouling time, and ${\hat{t}}_{\mathrm{foul}}^{-}$ and ${\hat{t}}_{\mathrm{foul}}^{+}$ fouling limit values as a function of measurement time (days). The dotted line corresponds to the maximum measurement time shown in Figure 4.

The estimated mean fouling time, ${\hat{t}}_{\mathrm{foul}}$, calculated as a function of number of measurements, *j*, fluctuates around a central value, which represents the mean value, $t_{\mathrm{foul}}$ (${\hat{t}}_{\mathrm{foul}}(t\to \infty )=t_{\mathrm{foul}}$). The two bounding values, ${\hat{t}}_{\mathrm{foul}}^{-}$ and ${\hat{t}}_{\mathrm{foul}}^{+}$, and the fouling limit values (**P** = 0.025 and **P** = 0.975) also vary over time. However, they gradually converge towards each other, since they are both directly linked to the standard deviation of the estimated fouling time (Equation (19)). This implies that, as the number of measurements increases, the estimated fouling range, $\left[{\hat{t}}_{\mathrm{foul}}^{-};{\hat{t}}_{\mathrm{foul}}^{+}\right]$, decreases. In Figure 5, ${\hat{t}}_{\mathrm{foul}}$ represents the extrapolated values of fouling time as a function of measurement time, while the dotted vertical line indicates the maximum duration of the measurement campaign (75 days).

### 4.5. Determination of Optimal Fouling Time

Up to this point, the analysis has focused on technical aspects. Once fouling occurs (at *t*_foul_), a systemic intervention—typically in the form of maintenance—must be performed within the estimated time interval. The time until maintenance, *t*_main_, whether measured from the start of operation or since the previous intervention, should fall within this interval, ${\hat{t}}_{\mathrm{foul}}^{\_}<t_{\mathrm{main}}<{\hat{t}}_{\mathrm{foul}}^{+}$. However, the exact timing of maintenance is not solely a technical decision—it must also consider economic factors.

Maintenance involves costs. The longer the interval between interventions, the fewer the maintenance events required per unit of time (e.g., per year). Since the number of fouling events, *n*_foul_, is inversely proportional to the time between interventions, *t*_main_, increasing *t*_main_ leads to fewer interventions, thus reducing maintenance frequency and cost—assuming fouling remains reversible. However, longer intervals also increase the risk of irreversible fouling, which results in significantly higher costs.

A distinction must be made between the maintenance costs incurred in a mild or reversibly fouled case and a severe or irreversibly fouled case. In the latter case, operational costs may increase further due to elevated energy consumption (e.g., higher pressure required to maintain flow rates as *TMP* increases). For simplicity, we assume that these costs are independent of other variables, and this is valid as long as the transmembrane pressure (*TMP*) does not significantly exceed the fouling threshold, *TMP*_foul_.

Let us denote the unit maintenance cost in the absence of fouling by *C*_0_, and the cost in the presence of fouling (including maintenance and potential refurbishment) by *C*_foul_. For this analysis, both costs are assumed to remain constant throughout the operational period. Accordingly, the annual operation cost as a function of the maintenance interval *t*_main_ can be expressed by the following relationship:

(22) $$C_{\mathrm{year}}=\frac{365}{t_{\mathrm{main}}}C_{0}+\frac{365}{t_{\mathrm{main}}}P(t\leq t_{\mathrm{main}})(C_{\mathrm{foul}}-C_{0}),$$

which consists of two opposing components: the first term represents cost reduction due to fewer interventions per unit time, and the second term represents cost increase, which is proportional to the probability of fouling **P** by the time of intervention.

By substituting Equation (20) into Equation (22) and rearranging terms, we obtain the following expression for the normalized annual cost as a function of the maintenance interval:

(23) $$\frac{C_{\mathrm{year}}}{C_{0}}=\frac{365}{t_{\mathrm{main}}}(1+\Phi (t=t_{\mathrm{main}})(\frac{C_{\mathrm{foul}}}{C_{0}}-1),$$

as $P(t\leq t_{\mathrm{main}})=\Phi (t=t_{\mathrm{main})}$. Introducing this parameter into Equation (23), we obtain the following equation:

(24) $$\lambda =100\left(\frac{C_{\mathrm{foul}}}{C_{0}}-1\right)\;\%,$$

which indicates what percentage of the maintenance cost is represented by the additional refurbishment cost due to fouling; that is, *λ* is the *refurbishment cost ratio*.

**Figure 6:**
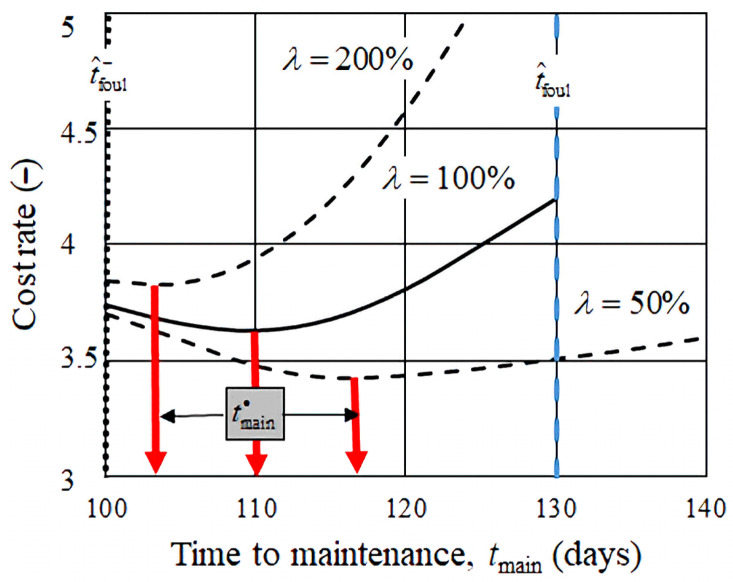
The normalized annual cost ratio *C*_year_/*C*_0_ as a function of the maintenance interval, *t*_main_, shown for different ratios of renovation to baseline maintenance costs (*λ* values: 50%, 100%, 200%). The solid black curve represents λ = 100%, while the dashed curves show λ = 50% and λ = 200%. The optimal maintenance interval $t_{\mathrm{main}}^{*}$ is indicated by red arrows. The blue dashed vertical line marks the estimated fouling time ${\hat{t}}_{\mathrm{foul}}$. Color coding (red and blue) is used to visually distinguish the cost-optimal point and the fouling limit, respectively.

Equation (23), expressed as a function of the parameter *λ*, yields a cost curve with a distinct minimum, where the time, $t_{\mathrm{main}}^{*}$, at which the minimum occurs, represents the economically optimal maintenance interval. As illustrated in Figure 6, increasing the value of λ causes the position of the minimum to shift towards the technical lower limit of the allowable maintenance interval. This reflects the growing emphasis on fouling-related costs relative to the baseline maintenance cost *C*_0_, resulting in a more conservative (earlier) intervention strategy.

### 4.6. Numerical Optimization

The standard approach for identifying the minimum of a function is to find the zero(s) of its first derivative. In this case, however, the resulting function does not permit an analytical solution. As a result, the optimal maintenance time *t*_main_ must be determined numerically. A practical approach is to use an iterative method based on the following condition, which defines the values of the minimum, i.e., the chosen, maintenance time $t_{\mathrm{mainl}}^{*}$ from the next relation, as follows:

(25) $$\frac{1}{t_{\mathrm{main}}}{\left.+\frac{\lambda }{t_{\mathrm{main}}}\Phi (\frac{t_{\mathrm{main}}-{\hat{t}}_{\mathrm{foul}}}{{\hat{s}}_{t_{\mathrm{foul}}}})\right|}_{t_{\mathrm{main}}^{*}}=\mathrm{MIN}.$$

Equation (25) can be solved, for example, using a step-by-step numerical method (e.g., the bisection method, the Newton–Raphson method, or a simple incremental search), as described in [38].

### 4.7. Interpretation of Probability Model

The probability that the membrane becomes fouled by the time of maintenance is given by the following equation:

(26) $$P_{\mathrm{foul}}=\Phi \left(\frac{t_{\mathrm{main}}^{*}-{\hat{t}}_{\mathrm{foul}}}{{\hat{s}}_{\mathrm{foul}}}\right).$$

Conversely, the probability that fouling has not occurred by the maintenance time is given by the following equation:

(27) $$P_{0}=1-P_{\mathrm{foul}}.$$

Equations (26) and (27) refer to the entire population of maintenance cycles. This means that, for *n* operating cycles, fouling is expected to occur in *n*_foul_ = *n***P**_foul_ cases, and not to occur in *n*_0_ = *n*P_0_ cases, where (*n* = *n*_foul_ + *n*_0_). In practice, for any given case, fouling either does or does not occur—there is no partial event. The occurrence of fouling can be inferred if, after maintenance, the transmembrane pressure (*TMP*) does not return to its initial value (*TMP*_0_) or within a two-standard-deviation confidence interval (see Equation (21)). The extent of the deviation from *TMP*_0_ can help determine whether significant fouling occurred and whether membrane renovation is required.

Finally, the remaining time until maintenance is one of the most important parameters for users, as it enables them to prepare for intervention:

(28) $$t_{\mathrm{back}}=t_{\mathrm{main}}^{*}-t.$$

### 4.8. Determination of Optimal Maintenance Time

To determine the optimal maintenance time, a dedicated computer program was developed. This tool can be used either in real time or as a predictive tool based on historical data. The primary quantity to be determined is the remaining time until maintenance. The quantity to be determined is the time remaining until maintenance. The program estimates the time remaining until maintenance, $t_{\mathrm{back}}$, based on the following calculation steps:

0. The fouling threshold transmembrane pressure, *TMP*_foul_, and the cost ratio, λ, are defined.
1. The program selects the incoming *TMP* data according to the filtering criteria described in Section 3.3 and arranges the data in a chronological order, up to the *jth* measurement taken at time *t_j_*.
2. For each *j* value (*t_j_* time), the program calculates the parameters of *a*, *SMT*_0_, and $s_{{\mathrm{TMP}}_{0}}$, in accordance with Equations (13) and (14) and then plots the lines defined by Equations (6) and (17).
3. The intersection points of these lines with the fouling threshold *TMP*_foul_ determine the estimated mean fouling time ${\hat{t}}_{\mathrm{foul}}$ and standard deviation ${\hat{s}}_{t_{\mathrm{foul}}}$, which in turn define the 95% confidence interval according to Equation (21).
4. Using these results, the distribution function Equation (20) is computed over the fouling interval with a time resolution of Δ*t* = 1 day.
5. With the known distribution, the normalized cost function Equation (23) is evaluated for each possible, *t*_main_.
6. The minimum of the cost function is then identified numerically, and the corresponding optimal maintenance time, $t_{\mathrm{main}}^{*}$, is selected. The program also calculates the remaining time until maintenance, *t*_back_; see Equation (28).

In the real-time application, the user interface presented in Figure 4 shows the following:

- The defined *TMP*_foul_;
- The selected *λ* value;
- The estimated mean fouling time ${\hat{t}}_{\mathrm{foul}}$;
- The standard deviation ${\hat{s}}_{t_{\mathrm{foul}}}$;
- The estimated optional maintenance time $t_{\mathrm{main}}^{*}$;
- The remaining time *t*_back_.

The estimation shown in Figure 6 was based on 75 days of recorded measurements (Figure 5). As operation continues, the procedure can be updated with new data. However, the calculated values of fouling time (both the mean and standard deviation) do not significantly change, provided that the average values of the key process parameters (e.g., feed water characteristics) remain constant. If these parameters change significantly over time, the reliability of the predictions may decrease. Improved accuracy can be achieved by restricting the analysis to the most recent 50–80 measurements, rather than the entire dataset.

## 5. Discussion

To verify the model, the selected dataset from Figure 3 (also shown in Figure 4) was used. The fitted parameters for Equation (13) are *a* = 0.020 bar/day and *TMP*_0_ = 10.5 bar. The standard deviations of the parameters from Equation (14) are as follows:

- $s_{a}$ = 0.002 bar/day.
- $s_{{\mathrm{TMP}}_{0}}$ = 0.265.

The standard deviation of the parameters from Equation (15) is as follows:

- $s_{\mathrm{TMP}}(t=75\;\mathrm{days})$ = 0.305.

Assuming a 25% permissible increase in the transmembrane pressure (*X* = 0.25) and using Equation (7), the critical fouling pressure is determined to be *TMP*_foul_ = 13.1 bar. Based on this, the extrapolated mean fouling time from Equation (18) is ${\hat{t}}_{\mathrm{foul}}$ = 130 days and the standard deviation from Equation (19) is ${\hat{s}}_{t_{\mathrm{foul}}}$ = 15 days. According to Equation (21), the corresponding confidence intervals are ${\hat{t}}_{\mathrm{foul}}^{-}$ = 100 days and ${\hat{t}}_{\mathrm{foul}}^{+}$ = 160 days as illustrated in Figure 5.

The technical maintenance window lies between 100 and 160 days. Within this range, the economically optimal intervention times depend on the assumed cost ratio (λ) as follows:

- For *λ* = 0.5 →$t_{\mathrm{main}}^{*}$ = 117 days;
- For *λ* = 1 → $t_{\mathrm{main}}^{*}$ = 110 days;
- For *λ* = 2 → $t_{\mathrm{main}}^{*}$ *=* 103 days.

In all cases, the optimal time is closer to the lower bound of the fouling interval.

The corresponding transmembrane pressures at this time have the following approximate values:

- 12.8 bar (22% increase);
- 12.7 bar (21%);
- 12.5 bar (19%).

The value of λ (renovation-to-maintenance cost ratio) is difficult to estimate, as cost data are often non-public or embedded within other operational expenses. Assuming λ to be in the range of 0.5–1.0, the economically optimal renovation time falls between 110 and 115 days.

However, this does not mean that exceeding this value is inherently incorrect—only that it is likely to result in increased cost—which may still be justified by operational constraints or strategic considerations. On the other hand, choosing $t_{\mathrm{main}}^{*}$ that is significantly higher than the fouling range could lead to the following:

- Increased operating pressure and energy costs;
- Reduced membrane lifespan;
- Irreversible fouling damage.

The fouling time estimates (${\hat{t}}_{\mathrm{foul}}$ mean, ${\hat{t}}_{\mathrm{foul}}^{+}$ maximal, and minimal ${\hat{t}}_{\mathrm{foul}}^{-}$) are calculated via extrapolation. As shown in Figure 5, these values stabilize after approximately 40–50 days of operation (*j* = 50–80). Beyond this point, further measurements only slightly affect the prediction. This implies that a reasonably accurate estimate can already be obtained after about 1–2 months, provided that the process conditions (e.g., feed water characteristics) remain stable. If conditions change—due to system renovation, raw water variation, or operating parameter shifts—then the model must be recalibrated, or its reliability may be compromised.

The estimated mean fouling time is ${\hat{t}}_{\mathrm{foul}}$= 130 days, while the standard deviation ${\hat{s}}_{t_{\mathrm{foul}}}$= 15 days. Whether this is considered high or low depends largely on the operational perspective. These values are primarily determined by the characteristics of the fluid being treated—such as its composition, turbidity, organic content, and variability. The influence of equipment parameters is secondary. Since the feed water quality is given in most applications and often cannot be altered, only limited control over the fouling behaviour is possible through equipment optimization.

However, this number cannot be increased simply by increasing the measurement frequency (e.g., from hourly to every 15 min), because consecutive measurements under unchanged conditions are not statistically independent. Instead, the number of effective, independent data points can only be increased by the following:

- Ensuring process variability is captured;
- Improving measurement accuracy, particularly of the transmembrane pressure (e.g., increasing resolution from 0.01 bar to 0.005 bar).

It must also be noted that, beyond 50–80 independent measurements, the improvement in estimation accuracy—especially the reduction in standard deviation—becomes marginal. Combining all factors, we estimate that the standard deviation of the fouling time *σ̂* could potentially be reduced by no more than 5 days under ideal conditions. Attempting further reduction would require disproportionately large effort or precision, which may not be justified from an operational or economic standpoint.

Given these limitations in improving accuracy, it is important to emphasize that the practical application of the model goes beyond prediction—it supports operational decision making and risk management. The model can be implemented in a software tool that predicts the expected fouling time for a specific system based on real-time or historical measurement data. When designing such a tool, several important factors must be considered, as follows:

- Fouling must be defined as a relative threshold, expressed as a dimensionless ratio *X*_foul_ (typically, 0.25), based on practical experience. While widely accepted, this threshold remains partly subjective and may vary depending on the system and operational goals.
- Because the relationship between *TMP* and time is stochastic, the critical fouling pressure *TMP*_foul_ must be defined with a reference to a probability level **P**. A higher probability level results in a shorter predicted fouling time and a more conservative intervention strategy.
- For a given fouling time, *t*_foul_, there is a direct relationship between *TMP*_foul_ and **P**: lowering *TMP*_foul_ increases the probability of remaining within safe operating limits.

Planning with a high fouling probability level may reduce the number of interventions but increases the risk of early fouling, unexpected downtime, and performance degradation. This is similar to the concept of Acceptable Quality Level (AQL) in industrial quality assurance: eliminating all defects is prohibitively expensive, while accepting a small, manageable defect rate is often more cost-effective. In membrane operation, the same logic applies, i.e., selecting the acceptable fouling probability is a risk management task, which must balance operational continuity against cost and system longevity. The model provides the quantitative foundation for such decisions, but the final determination must be made by the user, who best understands the operational context and system-specific trade-offs.

## 6. Summary

Membrane filtration technologies are widely used in water treatment around the world. However, due to their porous structure, membranes are susceptible to fouling, which reduces their efficiency and may ultimately render the membranes inoperable.

To maintain reliable operation, it is essential to anticipate fouling by predicting the expected fouling time. As fouling progresses, membrane resistance increases, leading to reduced flux. A critical flux threshold can be identified, beyond which maintenance or membrane replacement becomes necessary.

Under laboratory conditions, where operational parameters are known and well controlled, a deterministic relationship can be established between these parameters and the fouling time. In such cases, simple extrapolation—also referred to as point estimation—can be used to determine the fouling time. However, in real operational environments, parameter fluctuations and uncertainties are common. Consequently, the fouling time must be treated as a random variable, typically modelled with a normal distribution characterized by a mean value and standard deviation. Instead of point estimation, interval estimation becomes appropriate, defining a time range within which fouling is expected to occur with a given probability (e.g., 95%).

This implies the following:

- Below the lower bound (with **P** < 2.5%), fouling is unlikely;
- Above the upper bound (with **P** > 97.5%), fouling is highly likely.

This interval represents the technically valid fouling range, within which the maintenance time must be selected. The selection of the intervention time is based on minimizing the annual maintenance cost, accounting for both under- and over-maintenance risks.

The uniqueness of the presented model lies in its ability to determine not only a technically justified intervention time but also the economically optimal maintenance schedule based on probabilistic risk assessment.

## Data Availability

The datasets supporting the reported results of this study are not publicly available due to the confidential nature of the data, as they are based on a live industrial system. However, the general trends and results are presented in the figures and can be accessed in the form of graphical representations. The study is primarily based on model-based analysis, and the specific details of the raw data are not critical for understanding the conclusions.

## Glossary

**Symbol**	
*C*	cost (cost unit)
*J*	flux (L/(m^2^h))
*R*	resistance (m^−1^)
*TMP*	transmembrane pressure (bar)
*E_η_*	activation energy (kJmol^−1^)
*T*	temperature (K, °C)
*X*	ratio (-)
**P**	probability (-)
*R*	gas constant (8.84 Jmol^−1^K^−1^)
*a*	rate (barday^−1^)
*t*	time (day)
*s*	standard deviation (bar)
*i*	ordinal number of sample
*j*	sample number
*Φ*	standard distribution function (-)
*η*	viscosity (P, poise, water at 20 °C 0.01 P)
λ	cost rate
In the following list, the letter “*Q*” represents a general physical parameter (e.g., pressure, resistance, cost), and the subscripts (or superscripts, where applicable) indicate its specific contextual meaning. For example, *Q*ₘₐₓ refers to the maximum value of any such parameter. These notations are used consistently across the manuscript to express variations in a given quantity.	
*Q*_main_	value related to maintenance
*Q*_foul_	value under fouling conditions
*Q*_tot_	total value
*Q*_memb_	value related to membrane
*Q*_pol_	value related to polarization
*Q*_feed_	value related to feed conditions
*Q*_per_	value related to permeate
*Q*_TMP_	value related to transmembrane pressure
*Q*_0_	initial value
$\hat{Q}$	estimated value
$\bar{Q}$	average value
Q*	optimal value
$Q^{-}$	minimal value
$Q^{+}$	maximal value
